# Risk of mortality from diseases of the circulatory system due to occupational chronic radiation exposure, considering the radiation dose rate

**DOI:** 10.1038/s41598-026-43943-5

**Published:** 2026-03-24

**Authors:** Tamara Azizova, Evgeniya Grigoryeva, Nobuyuki Hamada

**Affiliations:** 1 Department of Radiation Epidemiology, Southern Urals Federal Research and Clinical Center for Medical Biophysics (SUFRCC MB), Ozyorsk, Russia; 2 Biology and Environmental Chemistry Division, Sustainable System Research Laboratory, Central Research Institute of Electric Power Industry (CRIEPI), Chiba, 270-1194 Japan

**Keywords:** Mortality from diseases of the circulatory system, Nuclear workers, Occupational chronic radiation exposure, Radiation dose rate, Diseases, Environmental sciences, Health care, Medical research, Risk factors

## Abstract

In this paper, we present findings of the analysis of mortality from diseases of the circulatory system in a cohort of Russian nuclear workers who were chronically exposed to ionizing radiation at radiation dose rate of 0.005–0.050 Gy/year. Excess relative risks of mortality from diseases of the circulatory system, cerebrovascular diseases, and ischemic stroke per unit total gamma-ray dose from external exposure accumulated at higher dose rates were found to be higher than the corresponding risks per unit total dose accumulated due to exposure at lower dose rates. Continuous radiation exposure at higher rates over a duration of 5 years was found to increase considerably the risk of mortality from diseases of the circulatory system, cerebrovascular diseases, and ischemic stroke.

**Supplementary Information:**

The online version contains supplementary material available at 10.1038/s41598-026-43943-5.

## Introduction

For improvement of the radiation protection system, it is essential to consider factors that could potentially affect and modify the dose-response relationship^1,2^. The dose rate is acknowledged to be among the key factors^3^. Experimental studies using animal models suggest that radiogenic risks vary with radiation dose rates^4,5^. Recently, it was demonstrated that in female rat models, the induction of benign tumors and malignant neoplasms in mammary tissue notably increased with higher radiation dose rates^6^. Meanwhile, studies evaluating the impact of radiation dose rate on health risks in cohorts of individuals exposed to ionizing radiation are limited and often produce controversial results^7–10^. In an earlier study of the mortality from ischemic heart disease (IHD) in the Russian cohort of nuclear workers of the Mayak facility, who had been chronically exposed to ionizing radiation, where radiation dose rates were considered to analyze the outcome, increased excess relative risks (ERR) of IHD mortality per unit of total absorbed gamma-ray dose from external exposure were observed at higher rates (in a range of 0.005–0.050 Gy/year) of dose accumulation^10^. Moreover, the magnitude of the IHD mortality risk estimate considerably increased with increasing duration of uninterrupted radiation exposure at higher dose rates. As such, whether the radiation dose rate in different exposure scenarios impacts health risks (for example, incidence of and mortality from cancer or non-cancer diseases) is still an open question, especially if other factors (e.g., age at exposure and time since exposure) are taken into account.

This study aimed to evaluate an ERR/Gy of mortality from cerebrovascular diseases (CeVD) including ischemic stroke (IS), and diseases of the circulatory system (DCS) per unit of the cumulative radiation dose from occupational chronic exposure considering the radiation dose rate and other factors, such as age at the start of radiation exposure, latent period, time since exposure, among others.

## Methods

### The study cohort and the follow-up period

This study was performed as a retrospective analysis of the cohort of employees of the Mayak Production Association, the first industrial nuclear enterprise in the former USSR. Located in the Southern Urals near the Ozyorsk city (a restricted-access area since its construction), the Mayak facility started its operation in 1948^11^. The analyzed cohort included all individuals hired at main Mayak facilities, including reactors, radiochemical and plutonium production plants, from 1 January 1948 to 31 December 1982, regardless of their sex, age, ethnicity, education level, social status, or other characteristics.

Cohort members were followed up from the date of hire at one of the main facilities until one of the following events occurred: the date of death, 31 December 2018 for those workers who were known to be alive and residing in Ozyorsk (residents); 31 December 2005 for those workers who were known to be alive but who had left Ozyorsk for another place of residence before 31 December 2005 (migrants), or the date of the last medical record for workers with unknown vital status and workers (migrants) who left Ozyorsk for another place of residence after 31 December 2005. It should be noted that the inconsistent end dates for follow-up between residents and migrants are due to the implementation of the Personal Data Protection Law in the Russian Federation on 31 December 2005, which made it impossible to collect information on migrants. As reported earlier^10^, all Mayak workers resided in Ozyorsk during their employment at the enterprise and received medical care from the same hospital. A migrant status was assigned to a worker based on a date when they left Ozyorsk for another place of permanent residency, as registered by special state service authorities.

It should be noted that for migrants, the only available source of information on causes of death was a death certificate issued by a civil registry office. In contrast, for residents, in addition to a death certificate, there were other information sources available. These included death certificates issued by a medical facility, forensic medical reports, autopsy reports, clinical records, and medical health files^12^. This variety of sources make it possible to analyze not only the underlying cause of death (e.g. CeVD) but also the contributing and immediate causes (e.g., IS).

The health outcomes analyzed for the entire cohort included mortality from DCS and from CeVD reported as the underlying cause of death (the International Classification of Diseases, 9th revision, ICD-9^13^: 390–459 and 430–438, respectively). Outcomes analyzed for the resident subcohort (RCS is the subcohort that included workers who lived in Ozyorsk and have never moved to another place of permanent residence) included mortality from ischemic stroke (IS), reported as a contributory or immediate cause of death for those individuals who died from CeVD (ICD-9: 434).

Radiation doses used in this analysis were individual estimates of annual doses of gamma rays and neutrons (from external exposure) and of alpha particles (from internal exposure) provided by the Mayak Worker Dosimetry System 2013 (MWDS-2013)^14,15^. As demonstrated in our previous studies^10,16^, adjusting for the alpha dose from internal exposure notably alters the excess relative risk of both incidence and mortality from DCS per unit of gamma dose from external exposure. Similar to previous studies^10,16^, in this analysis we used liver absorbed radiation doses as the MWDS-2013 did not provide radiation doses absorbed in organs and tissues of the circulatory system (e.g. in the heart and blood vessels). However, it is important to note that the biokinetic model used in MWDS-2013 consists of three main components: the respiratory tract model, the gastro-intestinal tract model, and the systemic model. The systemic model takes into account the plutonium metabolism in the liver and organs other than those included in the respiratory and gastro-intestinal tract models. All estimates of organ absorbed doses calculated with the systemic biokinetic model highly correlate in various organs (bone surface, red bone marrow, muscles, liver, gonads, kidneys, bladder walls) with a Pearson’s correlation coefficient of 0.99.

### Statistical analysis

The analyses were conducted for the entire cohort of Mayak workers (mortality from DCS and from CeVD) and the RSC (mortality from IS). For the analyses, we utilized the tabulated datasets (Table S1). The analysis was performed within the study cohorts.

For all analyses, we considered male and female workers together and applied a lag of 10 years to the radiation doses.

ERR/Gy estimates were calculated based on the Poisson’s regression using the AMFIT module of the EPICURE software^17^. To determine statistical significance, we computed 95% confidence intervals and p-values based on maximum likelihood using the AMFIT module. All statistical significance tests were two-sided. Differences were considered statistically significant (referred to as ‘significant’ hereafter) if the p-value was below 0.05 (*p* < 0.05).

First, in line with the previous study^16^, the ERR/Gy was calculated based on a conventional linear model that did not consider the radiation dose rate. Adjustments for the following factors were added via stratification: sex, attained age (< 20, 20–24, …, 80–84, ≥ 85), calendar period (1948–1950, 1951–1955, 1956–1960, …, 2011–2015, 2016–2018), smoking status (never smoked, ever smoked, unknown), alcohol consumption (seldom drinker, moderate drinker, alcohol abuse, unknown), migration status, and alpha dose from internal exposure. When adjusting for the alpha dose, workers who were not monitored for internal alpha-particle exposure were assigned to a separate category of “unknown” (all workers without bioassay measurements of alpha activity) rather than excluded from the dataset. Therefore, the Poisson regression model (hereinafter referred to as conventional model) was as follows:

1 $$\lambda = \lambda_0(S,\:aa,\:ct,\:smok,\:alc,\:mig,\:d\alpha)\cdot(1+\beta\cdot D\gamma),$$

where λ denotes a rate of mortality from DCS, CeVD, or IS, λ_0_ denotes a background rate of mortality from DCS, CeVD, and IS, s denotes sex, aa denotes attained age, ct denotes calendar period, smok denotes smoking status, alc denotes alcohol consumption, mig denotes migration status, dα denotes a category variable for cumulative liver-absorbed alpha dose from internal exposure (Gy), β denotes ERR/Gy, and Dγ denotes cumulative liver-absorbed gamma dose from external exposure (Gy).

Then, we carried out the analysis which considered the radiation dose rates defined using annual doses registered with individual dosimeters (Gy/year). In line with similar studies^10^, to evaluate the impact of the radiation dose rate on mortality from DCS, CeVD, and IS, we used a method of “dose windows”. 10 dose rate cut-off point were considered in the analysis. Cut-off points for the dose rate ranged from 0.005 to 0.050 Gy/year at 0.005 Gy interval. Table S2 gives an example of DγL and DγH calculations for cut-off point of 0.005 Gy/year^18^.

For each cut-off point, the model used in this analysis (hereinafter referred to as current model) was as follows:

2 $$\lambda = \lambda_0(s,\:aa,\:ct,\:smok,\:alc,\:mig,\:d\alpha)\cdot\left(1+\beta_L\cdot,D_{\gamma L}+\beta_H\cdot D_{\gamma H}\right)$$

where Dγ_L_ denoted total radiation dose accumulated at a rate below a defined cut-off value of the dose rate and Dγ_H_ denoted total radiation dose accumulated at a rate above a defined cut-off value of the dose rate, β_L_ denoted excess relative risk per unit of Dγ_L_ (ERR_L_/Gy) and β_H_ denoted excess relative risk Dγ_H_ (ERR_H_/Gy). To evaluate ERR/Gy, ERR_L_/Gy and ERR_H_/Gy for any cut-off point, we used a reference group consisting of workers chronically exposed to ionizing radiation at total doses 0–0.1 Gy. For estimation of ERR/Gy, we considered the total dose accumulated regardless of the dose rate. To evaluate ERR_L_/Gy, we considered the total dose accumulated at rates below a specified cut-off point, while to evaluate ERR_H_/Gy, we considered the total dose accumulated at a rate above that cut-off point. Comparison of the resulting estimates between the conventional and the present models was based on maximum likelihood.

Several sensitivity analyses were carried out to investigate the ERR/Gy further:

- Applying various lag periods (0, 5, 20, and 30 years) to radiation doses from occupational external and internal exposures. In this study, a lag period was a period before death during which radiation exposure was considered to have no impact on death occurrence. For all analyses with lag periods of x years, person-years from the beginning of employment were considered, while the first x years were assigned to the zero dose;
- Removing the adjustment for alpha dose from internal exposure;
- Analyzing a linear trend with the weighted combined gamma-neutron dose (based on the dose weighting factor for absorbed gamma dose of 1 and neutron dose of 10). To define the weighted combined gamma-neutron dose, the non-measured neutron dose was given a value of 0.00;
- Adding adjustments (via stratification) for additional factors: period of hire (1948–1958, 1959–1972, 1973–1982), age at hire (< 20, 20–29, ≥ 30);
- The effect of uninterrupted higher dose rate exposure during 5 years was assessed.

## Results

**Fig. 1:**
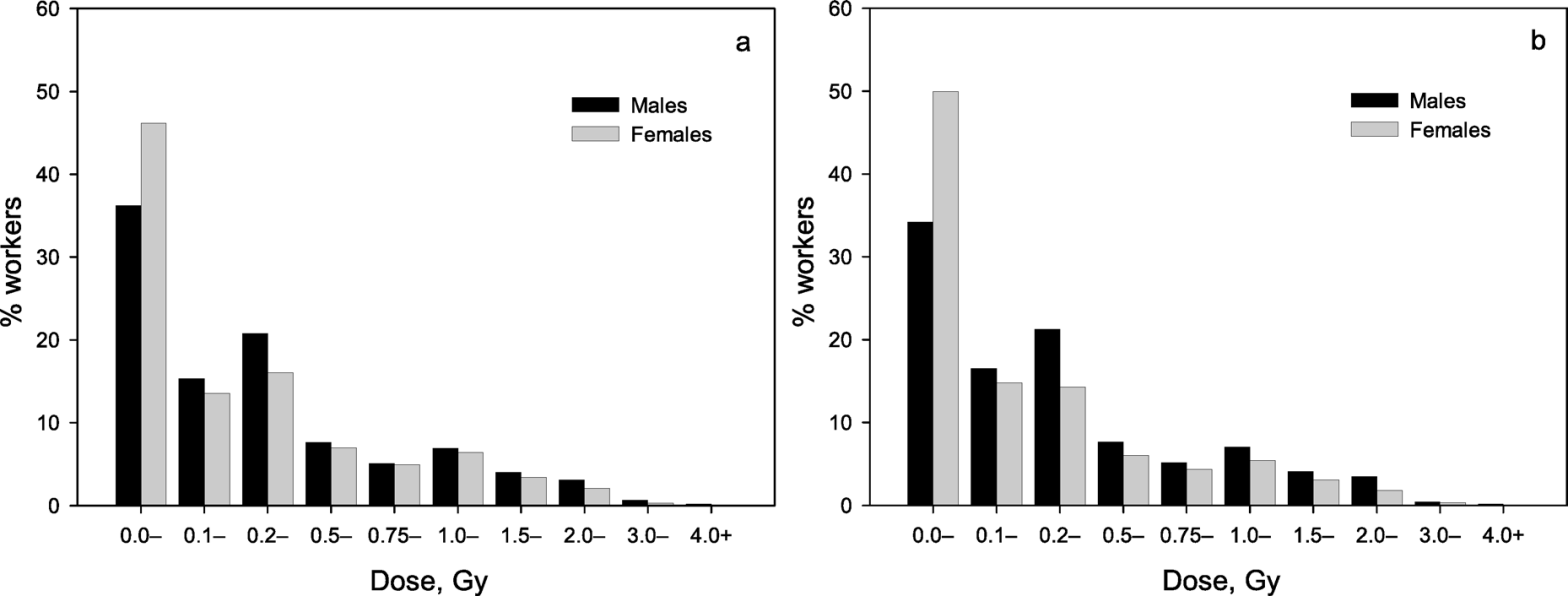
Distribution of workers of the entire cohort (a) and of the resident subcohort (b) by cumulative liver absorbed gamma dose from external exposure.

The study cohort included 22,377 workers, of whom 25.4% were females. The resident subcohort (RSC) consisted of 13,156 workers, with 27.9% being females. Forty-three workers who suffered from acute radiation sickness following acute gamma-neutron high dose-rate exposure were excluded from the cohort. Table 1 summarize major characteristics of the entire Mayak cohort and the RSC considered in this study. The majority of Mayak workers (76.1% of the entire study cohort and 76.2% of the RSC) were exposed to mixed radiation – externally to gamma rays and/or neutrons and internally to alpha particles emitted by incorporated plutonium. The remaining workers were exposed to ionizing radiation only from external sources. Figure 1 and Table S3 shows the distribution of workers in both the entire cohort and the RSC by gamma dose. It should be noted that the distributions of workers by gamma dose did not differ significantly between the entire cohort and the RSC (Pearson χ^2^ = 15.012; *p* = 0.09). At the end of the follow-up, mean total liver-absorbed gamma doses from external exposure (hereinafter, gamma doses (standard deviation)) were 0.43 (0.63) Gy for the entire cohort and 0.42 (0.60) Gy for the RSC.

**Table 1:** Main characteristics of the Mayak worker cohort.

Characteristic	Mayak Worker Cohort	Resident Subcohort (RSC)
Number of workers	22,377	13,156
Among them. females (%)^а^	5,689 (25.4%)	3,672 (27.9%)
Number of workers excluding those with ARS	22,334	13,124
Vital status confirmed at the end of the follow-up (%)^a^	21,337 (95.4%)	13,155 (99.9%)
Number of the deceased (%)^b^	14,362 (67.2%)	9,112 (69.3%)
Cause of death confirmed for the deceased (%)^c^	14,328 (99.8%)	9,096 (99.8%)
Age at death for the deceased, years (%)^c^ ≤ 20	16 (0.1%)	15 (0.2%)
21–30	318 (2.2%)	216 (2.4%)
31–40	572 (4%)	332 (3.6%)
41–50	1255 (8.7%)	734 (8.1%)
51–60	2926 (20.4%)	1765 (19.4%)
61–70	3995 (27.8%)	2410 (26.4%)
71–80	3567 (24.8%)	2327 (25.5%)
81–90	1546 (10.8%)	1182 (13.0%)
> 90	167 (1.2%)	131 (1.4%)
Mean age at death for the deceased (SD), years	64.6 (14.1)	65.4 (14.5)
Age at the end of the follow-up, years (%)^d^ ≤ 60	1150 (16.49%)	691 (17.09%)
61–70	2593 (37.18%)	1383 (34.21%)
71–80	2256 (32.34%)	1175 (29.06%)
81–90	874 (12.53%)	697 (17.24%)
> 90	102 (1.46%)	97 (2.4%)
Mean age at the end of the follow-up for alive cohort members (SD), years	75.51 (10.88)	71.21 (10.02)
Calendar period of hire, years (%)^a^ 1948–1958	12,297 (55.0%)	5,703 (43.3%)
1959–1972	6,606 (29.5%)	4,351 (33.1%)
1973–1982	3,474 (15.5%)	3,102 (23.6%)
Age at hire, years (%)^а^ ≤ 20	7,804 (34.9%)	4,431 (33.7%)
21–30	10,348 (46.2%)	5,792 (44%)
31–40	3,002 (13.4%)	2,093 (15.9%)
> 40	1,223 (5.5%)	840 (6.4%)
Mean age at hire (SD), years	24.9 (7.5)	25.5 (7.9)
Duration of employment, years (%)^а^ < 1	1,057 (4.7%)	201 (1.5%)
1–9	8,174 (36.5%)	1,706 (13%)
≥ 10	13,146 (58.8%)	11,249 (85.5%)
Mean duration of employment (SD), years	18.1 (14.3)	26.1 (12.9)
Number of workers exposed to mixed radiation (%)^a^	17,023 (76.1%)	3129 (76.2%)

ARS denotes acute radiation sickness;

SD denotes standard deviation;

^а^percentage of the cohort (subcohort);

^b^percentage of workers with confirmed vital status;

^c^percentage of the deceased workers;

^d^percentage of workers alive at the end of the follow-up.

**Fig. 2:**
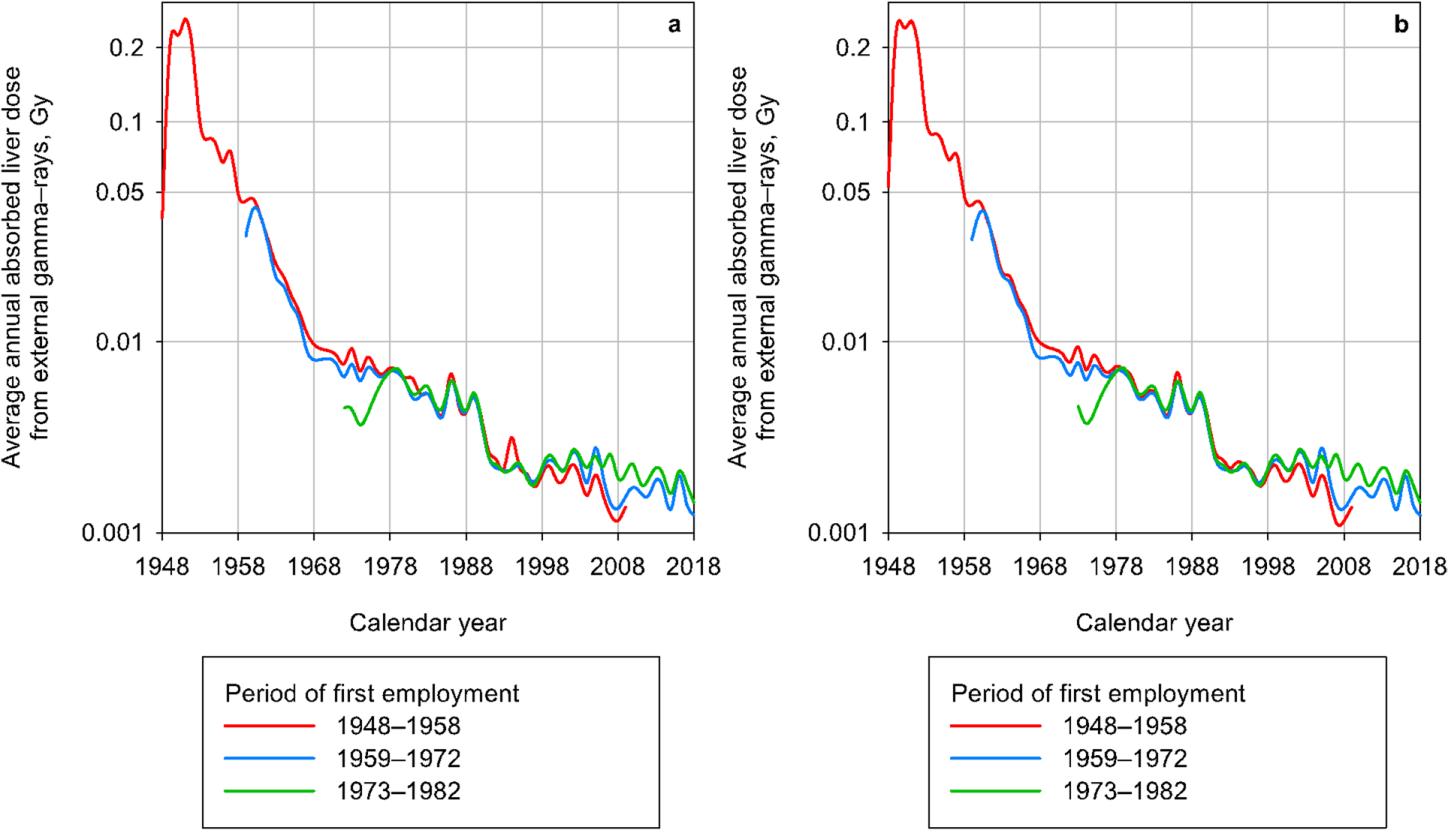
Change in the mean annual liver-absorbed gamma-ray dose from external exposure in the Mayak Worker cohort (panel **a**) and in the RSC (panel **b**) in relation to the calendar period.

As described earlier^10,11,16^, the working conditions during the early years of production activities at the enterprise were the most unfavorable, resulting in radiation exposure of the personnel at high levels of doses. Figure 2 shows the change in mean annual gamma doses for workers of the entire cohort and for those of the RSC in relation to the calendar period of hire at one of the main Mayak facilities. The analysis revealed that annual gamma doses from external exposure were significantly associated with the calendar period of hire. In 1951, the mean annual gamma dose was 0.25 Gy/year. Over the following decade, annual doses notably decreased to 0.05 Gy/year, and from 1960 to 1980, gamma doses continued to decline, eventually levelling off at 0.008 Gy/year. This trend in annual radiation doses was consistent for the entire cohort and for the RSC. Mean annual gamma doses were 0.05 (0.11) Gy for the entire cohort and 0.03 (0.08) Gy for the RSC.

It should be noted that workers of reactors and certain departments of the radiochemical and plutonium production facilities were exposed to neutrons (4,083 (18.2%) workers of the entire cohort, including 2,583 (19.6%) workers of the RSC). The distribution of the entire cohort workers and the RSC workers by the neutron dose is summarized in Table S3. The mean total liver-absorbed dose from neutrons was 0.0011 (0.0042) Gy for the entire cohort, and 0.0012 (0.0045) Gy for the RSC.

According to MWDS-2013, bioassay (24-hour urine samples) measurements of alpha activity from incorporated radionuclides were conducted only for 44.8% of the entire cohort workers and for 72.2% of the RSC workers who had been exposed to mixed radiation types. Table S3 displays the distribution of the entire cohort and RSC workers by alpha dose. The mean total liver-absorbed dose of alpha particles from internal exposure (referred to as ‘alpha dose’) was 0.25 (1.19) Gy for the entire cohort and 0.22 (1.12) Gy for the RSC.

At the end of the follow-up of the analyzed Mayak cohort, there were 6,019 recorded deaths from DCS as the underlying cause of death over 622,199 person-years. Among these, 3,481 (57.8%) were attributed to IHD, 1,808 (30.0%) due to CeVD, and 730 (12.2%) to other diseases. Additionally, it is important to note that, among members of the RSC, IS was reported as a contributing or immediate cause of death in 437 death records, which accounted for 37.4% of deaths from CeVD as an underlying cause.

Table 2 summarizes the results of the main analysis, which demonstrated significant differences in the mortality risk estimates for all analyzed diseases (DCS, CeVD, and IS) between the conventional model (which does not consider the dose rate) and the current one (which considers the dose rate). These differences were observed at all cut-off points except for 0.050 Gy/year for CeVD, and 0.005 Gy/year and 0.025–0.050 Gy/year for IS. No sex-related differences were found for any of the analyzed outcomes and any of the cut-off points (р>0.05; data not presented). It should be noted that at all cut-off points, estimates of ERR_H_/Gy (radiation exposure at dose rates higher than a cut-off point) were higher than ERR_L_/Gy (radiation exposure at dose rates below a cut-off point) for all outcomes. Moreover, the estimates of ERR_H_/Gy for DCS mortality were significant at cut-off values of 0.025–0.050 Gy/year. The corresponding estimates at any cut-off points were significant for IS mortality, but not significant for CeVD mortality (Table 2).

**Table 2:** Excess relative risks of mortality from DCS, CeVD, and IS in relation to the cumulative 10-year lagged liver-absorbed gamma dose from external exposure, adjusted for different non-radiation factors and the liver-absorbed alpha-particle dose from internal exposure (the main analysis: entire cohort).

Cut-off point (Gy)	Model parameters	DCS	CeVD	IS^а^
Without a cut-off point	ERR/Gy	0.03 (–0.02; 0.08)	0.03 (–0.05; 0.13)	0.23 (–0.00; 0.57)
0.005	ERR_L_/Gy	**–4.25 (–5.79; − 2.55)**	**–4.40 (–7.08; − 1.14)**	–5.26 (n/a; 0.50)
	ERR_H_/Gy	0.03 (–0.02; 0.08)	0.03 (–0.05; 0.13)	**0.22 (+ 0.00; 0.54)**
	*p* value ^b^	**< 0.001**	**0.01**	**0.049**
0.010	ERR_L_/Gy	**–2.14 (–2.83; − 1.40)**	**–2.01 (–3.17; − 0.63)**	**–2.56 (–4.39; − 0.00)**
	ERR_H_/Gy	0.04 (–0.01; 0.09)	0.04 (–0.04; 0.14)	**0.25 (0.03; 0.57)**
	*p* value ^b^	**< 0.001**	**0.005**	**0.034**
0.015	ERR_L_/Gy	**–1.54 (–2.00; − 1.03)**	**–1.52 (–2.31; − 0.59)**	**–2.02 (–3.34; − 0.26)**
	ERR_H_/Gy	0.04 (–0.01; 0.09)	0.05 (–0.03; 0.15)	**0.27 (0.04; 0.60)**
	*p* value ^b^	**< 0.001**	**0.002**	**0.015**
0.020	ERR_L_/Gy	**–1.15 (–1.50; − 0.76)**	**–1.15 (–1.76; − 0.42)**	–1.23 (–2.32; 0.24)
	ERR_H_/Gy	0.05 (–0.00; 0.10)	0.05 (–0.03; 0.15)	**0.28 (0.04; 0.61)**
	*p* value ^b^	**< 0.001**	**0.002**	**0.048**
0.025	ERR_L_/Gy	**–0.90 (–1.19; − 0.58)**	**–0.95 (–1.44; − 0.36)**	–0.85 (–1.75; 0.41)
	ERR_H_/Gy	**0.05 (+ 0.00; 0.10)**	0.06 (–0.03; 0.16)	**0.29 (0.04; 0.63)**
	*p* value ^b^	**< 0.001**	**0.002**	0.079
0.030	ERR_L_/Gy	**–0.68 (–0.94; − 0.40)**	**–0.75 (–1.18; − 0.23)**	–0.52 (–1.29; 0.58)
	ERR_H_/Gy	**0.05 (0.01; 0.11)**	0.06 (–0.03; 0.16)	**0.29 (0.04; 0.64)**
	*p* value ^b^	**< 0.001**	**0.004**	0.145
0.035	ERR_L_/Gy	**–0.51 (–0.74; − 0.26)**	**–0.65 (–1.02; − 0.20)**	–0.55 (–1.19; 0.38)
	ERR_H_/Gy	**0.06 (0.01; 0.11)**	0.06 (–0.02; 0.16)	**0.31 (0.05; 0.67)**
	*p* value ^b^	**< 0.001**	**0.004**	0.076
0.040	ERR_L_/Gy	**–0.40 (–0.60; − 0.17)**	**–0.51 (–0.85; − 0.10)**	–0.49 (–1.07; 0.34)
	ERR_H_/Gy	**0.06 (0.01; 0.11)**	0.06 (–0.03; 0.17)	**0.32 (0.06; 0.69)**
	*p* value ^b^	**< 0.001**	**0.011**	0.068
0.045	ERR_L_/Gy	**–0.28 (–0.46; − 0.07)**	–0.37 (–0.69; 0.01)	–0.29 (–0.83; 0.49)
	ERR_H_/Gy	**0.05 (+ 0.00; 0.11)**	0.06 (–0.03; 0.16)	**0.31 (0.05; 0.69)**
	*p* value ^b^	**0.004**	**0.035**	0.141
0.050	ERR_L_/Gy	–0.19 (–0.37; +0.00)	–0.25 (–0.55; 0.12)	–0.19 (–0.71; 0.55)
	ERR_H_/Gy	**0.05 (+ 0.00; 0.10)**	0.05 (–0.04; 0.16)	**0.31 (0.04; 0.69)**
	*p* value ^b^	**0.023**	0.117	0.193

Values in bold are statistically significant estimates and differences;

The dataset was stratified by sex, attained age, calendar period, smoking status, alcohol consumption, and alpha-particle dose;

ERR/Gy denotes the excess relative risk per unit of radiation dose;

DCS denotes diseases of the circulatory system (ICD-9 codes: 390–459);

CeVD denotes cerebrovascular diseases (ICD-9 codes: 430–438);

IS denotes ischemic stroke (ICD-9 code: 434);

n/a denotes the undefined boundary of the confidence interval.

^a^The analyzed dataset is limited to residents.

^b^Maximum likelihood test for comparison between models with and without a cut-off point.

Tables S4, S5 and S6 summarize the results for the mortality risk of DCS, CeVD, and IS in relation to the radiation dose rate, using various lag periods (0, 5, 20, and 30 years). It should be noted that differences in the DCS mortality risk in the analyzed cohort between the conventional and current models were statistically significant at all cut-off points across all lag periods (0, 5, 20, 30 years), as observed in the main analysis (10-year lag) (Table S4). A similar pattern was seen for CeVD mortality, although there were some exceptions at specific cut-off points with 20- and 30-year lags (Table S5). In the analysis of the IS mortality risk, there were significant differences between the conventional and current models at all cut-off points but only with 0, 5, and 10-year lag periods, except for 0.050 Gy/year with 0-year lag (Table S6). DCS mortality risks due to radiation exposure at dose rates above a cut-off point (ERR_H_/Gy) were found to be higher compared to ERR_L_/Gy and were statistically significant across all lag-periods at cut-off points of 0.020–0.050 Gy/year (Table S4). ERR_H_/Gy of total radiation dose for CeVD mortality were also higher than ERR_L_/Gy, but this was statistically non-significant for all cut-off points across all lag periods (Table S5). The analysis of the IS mortality risk in the RSC revealed higher estimates of ERR_H_/Gy of the total radiation dose than ERR_L_/Gy, which were statistically significant for all cut-off points across all lag periods, except for 0.005 Gy/year with 0- and 20-year lag periods (Table S6). It should be noted that the magnitude of ERR_H_/Gy and ERR_L_/Gy for DCS mortality remained stable with the extension of the lag period for all cut-off points. The corresponding estimates of ERR_H_/Gy and ERR_L_/Gy for CeVD mortality increased by 15%–25% from 0-year lag by the 30-year lag, and a considerable risk increase of 40%–50% was observed for IS mortality with the 5-year lag period.

The sensitivity analysis of DCS, CeVD, and IS mortality risks, which included the weighted total absorbed gamma and neutron doses from external exposure (with the weighting factor of 10), yielded results that were similar to those of the main analysis (Tables S7, S8, and S9). Meanwhile, removing the adjustment for alpha dose from the model led to a considerable decrease in the ERR_H_/Gy and even in a loss of the statistical significance for DCS and IS (by 30–40% and 60–80%, respectively) (Tables S7 and S9). For CeVD, the ERR_H_/Gy decreased by 60–80% without affecting the statistical significance (Table S8). On the contrary, eliminating the alpha-dose adjustment did not notably affect the risk of mortality from the diseases of interest due to exposure at dose rates below any of the cut-off points, nor did it change the significance of the risk estimates (Tables S7, S8 and S9).

After incorporating additional adjustments for the period of or age at hire into the model, significant differences in DCS and CeVD mortality risks were also observed between the conventional and the current models at all cut-off points (except for 0.050 Gy/year for CeVD). These findings are in good agreement with the results of the main analysis (Table 2, S7, and S8). Meanwhile, the current analysis of IS mortality risks in the RSC showed the estimates significantly different from those obtained with the conventional model only at certain cut-off points – again, consistent with the main analysis (Table 2 and S9).

Including an adjustment for age at hire in the model resulted in an increase in the ERR_H_/Gy for mortality from DCS, CeVD, and IS, and the risk achieved statistical significance at all cut-off points (which is consistent with the main analysis). Meanwhile, the ERR_L_/Gy modestly decreased, except at the cut-off point of 0.050 Gy/year for CeVD and IS (Table 2, S7, S8, and S9).

Including an adjustment for period of hire in the model led to a modest increase in the ERR_H_/Gy for mortality from DCS, CeVD, and IS, and the risk estimates achieved statistical significance for all cut-off points (except 0.005 Gy/year for CeVD). Meanwhile, the ERR_L_/Gy decreased, except at the cut-off point of 0.050 Gy/year for CeVD and IS (Table 2, S7, S8, and S9).

We also conducted an analysis to examine the impact of continuous radiation exposure at dose rates above specified cut-off points over an extended period (five years) on the risks of the analyzed outcomes (Table 3). The continuous radiation exposure at dose rates above cut-off points over five years considerably increased the risk of mortality from all analyzed outcomes (DCS, CeVD, and IS) compared to the risk estimated for exposures lasting only one year at dose rates above the cut-off points (Tables 2 and 3).

**Table 3:** Excess relative risks of mortality from DCS, CeVD, and IS in relation to the cumulative 10-year lagged liver-absorbed gamma dose from external exposure, adjusted for different non-radiation factors (sensitivity analysis: continuous radiation exposure at doses above a cut-off point over five years, both sexes, entire cohort).

Cut-off point (Gy)	Model parameters	Duration of radiation exposure at doses above a cut-off point over minimum 5 years		
		DCS	CeVD	IS^а^
Without the cut-off point	ERR/Gy	0.03 (–0.02; 0.08)	0.03 (–0.05; 0.13)	0.23 (–0.00; 0.57)
0.005	ERR_L_/Gy	–0.01 (–0.07; 0.05)	–0.01 (–0.10; 0.11)	0.12 (–0.17; 0.54)
	ERR_H_/Gy	**0.15 (0.03; 0.30)**	0.15 (–0.08; 0.42)	0.43 (–0.06; 1.09)
	*p* value ^b^	**0.039**	0.291	0.382
0.010	ERR_L_/Gy	–0.02 (–0.07; 0.04)	–0.02 (–0.11; 0.10)	0.06 (n/a; 0.45)
	ERR_H_/Gy	**0.20 (0.06; 0.35)**	0.20 (–0.04; 0.49)	**0.58 (0.04; 1.32)**
	*p* value ^b^	**0.007**	0.149	0.156
0.015	ERR_L_/Gy	–0.03 (–0.08; 0.03)	–0.03 (–0.12; 0.08)	0.09 (–0.17; 0.48)
	ERR_H_/Gy	**0.24 (0.09; 0.40)**	0.26 (–0.00; 0.57)	**0.56 (+ 0.00; 1.31)**
	*p* value ^b^	**0.002**	0.066	0.212
0.020	ERR_L_/Gy	–0.03 (–0.08; 0.03)	–0.04 (–0.13; 0.07)	0.05 (na; 0.42)
	ERR_H_/Gy	**0.29 (0.14; 0.47)**	**0.35 (0.06; 0.69)**	**0.71 (0.08; 1.56)**
	*p* value ^b^	**< 0.001**	**0.022**	0.104
0.025	ERR_L_/Gy	–0.02 (–0.07; 0.04)	–0.02 (–0.11; 0.10)	0.08 (–0.17; 0.45)
	ERR_H_/Gy	**0.28 (0.11; 0.46)**	0.26 (–0.04; 0.62)	**0.70 (0.03; 1.61)**
	*p* value ^b^	**0.002**	0.128	0.147
0.030	ERR_L_/Gy	–0.02 (–0.07; 0.04)	–0.02 (–0.11; 0.09)	0.04 (na; 0.40)
	ERR_H_/Gy	**0.30 (0.12; 0.49)**	0.30 (–0.02; 0.69)	**0.89 (0.15; 1.91)**
	*p* value ^b^	**0.002**	0.091	0.063
0.035	ERR_L_/Gy	–0.01 (–0.06; 0.05)	–0.02 (–0.11; 0.09)	0.06 (–0.17; 0.43)
	ERR_H_/Gy	**0.26 (0.08; 0.47)**	0.31 (–0.03; 0.72)	**0.90 (0.10; 2.00)**
	*p* value ^b^	**0.01**	0.098	0.088
0.040	ERR_L_/Gy	–0.01 (–0.06; 0.05)	–0.01 (–0.10; 0.10)	0.12 (–0.14; 0.49)
	ERR_H_/Gy	**0.28 (0.08; 0.50)**	0.27 (–0.08; 0.70)	0.75 (–0.10; 1.92)
	*p* value ^b^	**0.009**	0.174	0.231
0.045	ERR_L_/Gy	–0.00 (–0.05; 0.06)	–0.01 (–0.10; 0.10)	0.11 (–0.14; 0.47)
	ERR_H_/Gy	**0.24 (0.04; 0.46)**	0.30 (–0.06; 0.74)	0.87 (–0.04; 2.13)
	*p* value ^b^	**0.032**	0.141	0.166
0.050	ERR_L_/Gy	–0.00 (–0.05; 0.06)	–0.01 (–0.10; 0.10)	0.10 (–0.14; 0.45)
	ERR_H_/Gy	**0.24 (0.04; 0.47)**	0.35 (–0.03; 0.82)	**1.01 (0.03; 2.41)**
	*p* value ^b^	**0.035**	0.099	0.117

Values in bold are statistically significant estimates and differences;

The dataset was stratified by sex, attained age, calendar period, smoking status, alcohol consumption, and alpha-particle dose;

ERR/Gy denotes the excess relative risk per unit of radiation dose;

DCS denotes diseases of the circulatory system (ICD-9 codes: 390–459);

CeVD denotes cerebrovascular diseases (ICD-9 codes: 430–438);

IS denotes ischemic stroke (ICD-9 code: 434);

n/a denotes the undefined boundary of the confidence interval.

^a^The dataset was limited to residents.

^b^Maximum likelihood test for comparison between models with and without a cut-off point.

## Discussion

ICRP Publication 118^19^ suggested that the risk of radiation-induced effects in tissues with slow metabolism, like the heart, is largely affected by the dose rate. However, this suggestion still needs further validation, especially concerning the effects resulting from chronic exposures^9,10^.

In this retrospective study, we evaluated the impact of radiation dose rates (based on individually measured annual radiation doses) that ranged from 0.005 Gy/year to 0.050 Gy/year on mortality from DCS, CeVD, and IS in the Russian cohort of Mayak workers, who were chronically exposed to radiation during their professional activities. As noted above, cohort studies designed to assess the effects of radiation dose rates during chronic exposures are rather sparse^7–10^. The Mayak worker cohort is one of the few suitable for this type of analysis, primarily thanks to the availability of individual estimates of gamma-ray doses from external exposure. These doses were measured using personal film badges throughout each worker’s employment period. Moreover, this cohort also has data on individual estimates of alpha-particle doses from internal exposure, based on bioassay measurements of alpha activity and calculated using biokinetic and dosimetry models. This cohort also has complete (99.8%) and high-quality data on causes of death, with autopsy examinations conducted for 33.8% of its members. Furthermore, the cohort of Mayak workers is numerous, including 22,377 individuals, and has a long follow-up period which exceeds 70 years.

In previous studies of DCS mortality, we used a linear model with adjustments for non-radiation factors (sex, attained age, calendar period, alcohol consumption, smoking status, and migration status while considering the entire cohort in the dataset for analysis) and alpha dose^16^. That analysis did not show a significant association with the total liver-absorbed gamma dose from external exposure for either DCS (ERR/Gy = 0.03; 95% confidence intervals (CI) -0.02, 0.08), or CeVD (ERR/Gy = 0.03; 95% CI: -0.05, 0.13), or IS (ERR/Gy = 0.23; 95% CI: -0.00, 0.57).In the current study, the ERR/Gy of total gamma dose from external exposure for mortality from DCS and CeVD in the entire cohort and for the IS in the RSC were calculated using the model that considered the radiation dose rate (Gy/year).

This study revealed significant differences in risk estimates for mortality from the analyzed diseases (DCS, CeVD, and IS) between the conventional model that does not account for the radiation dose rate and the current model that does. Additionally, the observed ERR/Gy of mortality from DCS, CeVD, and IS per unit of gamma dose from external exposure were higher when the dose was accumulated at higher rates (exceeding cut-off values) compared to dose accumulation at lower rates (below cut-off points). These findings are consistent with earlier analyses of IHD conducted in the same Mayak worker cohort^10^ and in a cohort of Hanford nuclear workers^9^, which also indicated an increased mortality risk from IHD associated with higher dose-rate exposures to ionizing radiation. We did not find any significant difference in risk estimates for males and females for either outcome at any of the cut-off points, even though the levels of the estimates varied. This was likely due to the varying statistical power of the separate analyses.

In this study, we investigated whether the risk of mortality from DCS, CeVD, and IS estimated in relation to the radiation dose rate is modified by other factors, such as another type of radiation exposure (internal alpha-particle exposure from incorporated plutonium), latency (lag period), the age at and calendar period of hire.

Important to note that the Mayak worker cohort has a very complex dosimetric characteristics. The workers of the study cohort experienced combined radiation exposure – they were exposed externally to gamma rays and internally to alpha particles from plutonium simultaneously. Moreover, a small percentage (18%) of these workers were exposed to neutrons externally. The external exposure (gamma-ray and neutron) was experienced by a worker at a workplace (during 6–8 h long shifts) while internal alpha exposure was continuous and prolonged since alpha-emitting nuclides entered their body. It is worth noting that external radiation exposure (gamma-ray and neutron) stops once a worker leaves the company, while internal alpha exposure persists until death. As reported earlier^10^, some Mayak workers were internally exposed to radionuclides other than plutonium; however, the contribution of plutonium to the total alpha dose in the Mayak worker cohort was the most substantial (> 90%).

It is important to highlight that the removing the alpha-dose adjustment from the model resulted in a decrease in the ERR_H_/Gy for all analyzed diseases, while the ERR_L_/Gy estimates remained stable. In previous analyses using the conventional model that did not account for the radiation dose rate, adjustments for alpha-particle dose (removing or adding it to the model) affected the risk of incidence of and mortality from IHD and CeVD, as well as mortality from DCS, in the cohort of Mayak workers^16,20,21^. When employing the model that considered the radiation dose rate, the IHD mortality risk in this cohort was also modified^10^. The authors argue that it is essential to consider all types of radiation exposures when analyzing cohorts of individuals exposed to mixed radiation (internally and externally). Failing to do so may lead to an overestimation of risk, especially with models that take into account not only the cumulative radiation dose but also the radiation dose rate.

The age at which a person is exposed to ionizing radiation (in the analyzed cohort, the age they are hired at a nuclear facility or the age when they begin to experience chronic radiation exposure) is among the factors modifying the radiogenic risk estimate. Epidemiological studies indicate that the excess relative risk per unit of cumulative radiation dose decreases as the age at exposure increases^22–25^. Previous analyses of IHD among Mayak nuclear workers conducted using the conventional model that did not account for the radiation dose rate also revealed a significant decreasing trend in risk with advancing age at exposure. This trend was linked to the differing sensitivity of various age groups to ionizing radiation. Additionally, this study demonstrates that the age at which individuals begin experiencing chronic radiation exposure (the age at hire at the Mayak PA) notably affects the ERR/Gy estimate. It should also be noted that adding an adjustment for the calendar period of hire to the model results in an increase in the ERR/Gy (in case of the conventional model) and in the ERR_H_/Gy (in case of the current model which accounts for the radiation dose rate) while the ERR_L_/Gy decreases. This result likely arises from the fact that, in the analyzed cohort, the period of hire correlates with both the cumulative radiation dose (the Pearson’s correlation coefficient of -0.41 (*p* < 0.001)) and the radiation dose rate (the Pearson’s correlation coefficient of -0.32 (*p* < 0.001)), meanwhile there is no correlation between the period of hire and the age at hire (the Pearson’s correlation coefficient of 0.00 (*p* = 0.291)).

In this study, as well as in previous research^10,16^, we observed that lagging of the gamma dose changed the ERR/Gy estimates. In general, the changes followed a similar pattern, except for the ERR_H_/Gy for CeVD mortality which was not significant for either lag period. Probably, this finding can be explained with the fact that an underlying condition for all analyzed diseases is atherosclerosis in various blood vessels, which is a slowly progressing, multi-stage systemic process that involves the formation of atherosclerotic plaques and the complications caused by their rupture. Several studies suggest that the ionizing radiation can initiate and/or promote this multifactorial process, modifying almost all known stages of its pathogenesis^19,26–31^. And the question about the optimal lag periods most suitable for analyzing the risk of non-cancer health effects due to chronic radiation exposure remains open. Importantly, it should be highlighted that continuous radiation exposure over five years at higher dose rates considerably increased the excess relative risk per unit of cumulative radiation dose from chronic exposure for all analyzed outcomes (DCS, CeVD, and IS) and IHD^10^.

The present results contribute to the update of the radiogenic risk of mortality from DCS and can be used for improvements of the radiological protection recommendations.

## Supplementary Information

Below is the link to the electronic supplementary material.

Supplementary Material 1

## Data Availability

This was a retrospective epidemiological study that did not involve direct participation of human subjects and was based on depersonalized data maintained in the database of the Southern Urals Federal Research and Clinical Center for Medical Biophysics of the Federal Medical Biological Agency, formerly the Southern Urals Biophysics Institute (SUBI). The dataset is the intellectual property of the Southern Urals Federal Research and Clinical Center for Medical Biophysics of the Federal Medical Biological Agency, Ozyorsk, Chelyabinsk Region, 456780, Russia. For privacy reasons, the data are not publicly available. These restrictions are imposed by Federal Act No. 323 (21 November 2011) on the Basics of Health Care for Russian Citizens and Federal Act No. 152 (27 July 2014) on Personal Data. Access to the Mayak Worker Cohort data requires approval from the Institutional Ethics Review Board of the Southern Urals Federal Research and Clinical Center for Medical Biophysics of the Federal Medical Biological Agency. Requests for access should be directed to Dr. Tamara Azizova (Head, Radiation Epidemiology Department) or Dr. Yuliya Tsareva (Researcher, Radiation Epidemiology Laboratory, member of the Institutional Ethics Review Board; tsareva@subi.su).
