# Supplementary material for: Risk of mortality from diseases of the circulatory system due to occupational chronic radiation exposure, considering the radiation dose rate
Source: Sci Rep. 2026 Mar 24;16:14797. doi: 10.1038/s41598-026-43943-5 (PMC13168313; doi:10.1038/s41598-026-43943-5)
Supplement: Supplementary file 1 — Supplementary Material 1 [file 41598_2026_43943_MOESM1_ESM.docx]

**RISK OF MORTALITY FROM DISEASES OF THE CIRCULATORY SYSTEM DUE TO OCCUPATIONAL CHRONIC RADIATION EXPOSURE, CONSIDERING THE RADIATION DOSE RATE**

SUPPLEMENTARY MATERIAL

Table S1. Variables used in the model for analyses

| Type | Variable | Description | |
| --- | --- | --- | --- |
| Fixed | Sex | 1: M | 2: F |
| Fixed | Period of hire | 1: 1948–1958  2: 1958–1972 | 3: 1973–1982 |
| Fixed | Age at hire | 1: <20  2: 20–25 | 3: 25–30  4: ≥30 |
| Fixed | Smoking status | 1: never smoker  2: ex-smoker | 3: smoker  4: unknown |
| Fixed | Alcohol drinking habit status | 1: never-drinker  2: seldom-drinker  3: moderate-drinker | 4: heavy-drinker  5: unknown |
| Fixed | Migration status | 1: residents  2: migrants | 3: unknown |
| Fixed | Duration of employment (year) | 1: <1  2: ≥1 |  |
| Time-dependent | Attained age | 1: <20  2: 20–25  3: 25–30  4: 30–35  5: 35–40  6: 40–45  7: 45–50  8: 50–55 | 9: 55–60  10: 60–65  11: 65–70  12: 70–75  13: 75–80  14: 80–85  15: ≥85 |
| Time-dependent | Calendar period | 1: 1948–1950  2: 1951–1955  3: 1956–1960  4: 1961–1965  5: 1966–1970  6: 1971–1975  7: 1976–1980 | 8: 1981–1985  9: 1986–1990  10: 1991–1995  11: 1996–2000  12: 2001–2005  13: 2006–2010  14: 2011–2015  15: 2016–2018 |
| Time-dependent | Liver-absorbed gamma dose from external exposure (Gy), lagged for 0, 5, 10, 20, 30 years | 1: 0–0.1  2: 0.1–0.2  3: 0.2–0.5  4: 0.5–0.75  5: 0.75–1.00 | 6: 1.00–1.50  7: 1.50–2.00  8: 2.00–3.00  6: ≥3.00 |
| Time-dependent | Cumulative liver-absorbed gamma dose from external exposure at a rate below a specified cut-off point of a dose-rate window (Gy), lagged for 0, 5, 10, 20, 30 years | 1: 0–0.1  2: 0.1–0.2  3: 0.2–0.5  4: 0.5–0.75  5: 0.75–1.00 | 6: 1.00–1.50  7: 1.50–2.00  8: 2.00–3.00  6: ≥3.00 |
| Time-dependent | Cumulative liver-absorbed gamma dose from external exposure at a rate above a specified cut-off point of a dose-rate window (Gy), lagged for 0, 5, 10, 20, 30 years | 1: 0–0.1  2: 0.1–0.2  3: 0.2–0.5  4: 0.5–0.75  5: 0.75–1.00 | 6: 1.00–1.50  7: 1.50–2.00  8: 2.00–3.00  6: ≥3.00 |
| Time-dependent | Liver-absorbed alpha dose from internal exposure (Gy), lagged for 0, 5, 10, 20, 30 years | 1: unmeasured  2: 0.00–0.025  3: 0.025–0.05  4: 0.05–0.1  5: 0.1–0.15 | 6 0.15–0.25  7: 0.25–0.5  8: 0.5 –1.00  9: ≥1.00 |
| Time-dependent | Weighted sum of liver-absorbed gamma and neutron dose (Sv), lagged for 10 years | 1: 0–0.1  2: 0.1–0.2  3: 0.2–0.5  4: 0.5–0.75  5: 0.75–1.00 | 6: 1.00–1.50  7: 1.50–2.00  8: 2.00–3.00  6: ≥3.00 |
| Time-dependent | Cumulative weighted sum of liver absorbed gamma and neutron dose (Sv) at a rate below a specified cut-off point of a dose-rate window (Gy), lagged for 0, 5, 10, 20, 30 years | 1: 0–0.1  2: 0.1–0.2  3: 0.2–0.5  4: 0.5–0.75  5: 0.75–1.00 | 6: 1.00–1.50  7: 1.50–2.00  8: 2.00–3.00  6: ≥3.00 |
| Time-dependent | Cumulative weighted sum of liver absorbed gamma and neutron dose (Sv) at a rate above a specified cut-off point of a dose-rate window (Gy), lagged for 0, 5, 10, 20, 30 years | 1: 0–0.1  2: 0.1–0.2  3: 0.2–0.5  4: 0.5–0.75  5: 0.75–1.00 | 6: 1.00–1.50  7: 1.50–2.00  8: 2.00–3.00  6: ≥3.00 |
| Estimated | Attained age | Person-years weighted by mean age (in years) | |
|  | Cumulative liver-absorbed gamma dose from external exposure (Gy), lagged for 0, 5, 10, 20, 30 years | Person-years weighted by cumulative liver-absorbed gamma dose lagged for 0, 5, 10, 20, 30 years | |
|  | Cumulative weighted sum of liver-absorbed gamma and neutron dose (Gy), lagged for 10 years | Person-years weighted by cumulative weighted sum of liver absorbed gamma and neutron doses lagged for 10 years | |
|  | Cumulative liver-absorbed alpha dose from internal exposure (Gy), lagged for 0, 5, 10, 20, 30 years | Person-years weighted by cumulative liver-absorbed alpha dose lagged for 0, 5, 10, 20, 30 years | |
|  | Person-years | at risk | |
|  | Deaths | 1: DCS: ICD-9 codes 390–459  1: CeVD: ICD-9 codes 430–438  1: IS: ICD-9 codes 434 | |

Data on smoking habits were taken into account over the entire follow-up period and estimated with qualitative and quantitative indices. The qualitative index included values ‘unknown’, ‘never smoker’, ‘ex-smoker’ and ‘smoker’. ‘Never smoker’ was assumed to be a worker who reported during a series of annual mandatory medical examinations that he/she had never smoked. For stratification purposes, categories ‘ex-smoker’ and ‘smoker’ were combined to build one category ‘ever smoker’.

Data on the alcohol drinking habit were also taken into account over the entire follow-up period and estimated only with a qualitative parameter with values ‘unknown’, ‘seldom-drinker’, ‘moderate-drinker’ and ‘heavy-drinker’. A ‘seldom-drinker’ was assumed to be a worker who reported during a series of annual mandatory medical examinations that he/she had never drunk alcohol or drank seldomly only small amounts. A ‘moderate drinker’ was assumed to be a worker who stated during mandatory health check-ups that he/she drank moderately. A ‘heavy drinker’ was assumed to be a worker whose medical files contained information on incidents of abusive drinking behavior, inebriety or chronic alcoholism.

Table S2. Illustration of two dose-rate windows (for instance: cut point x = 5 mGy/year) [1]

| Dose | | Year | | | | | | | |
| --- | --- | --- | --- | --- | --- | --- | --- | --- | --- |
|  |  | 1971 | 1972 | 1973 | 1974 | 1975 | 1976 | 1977 | 1978 |
| Annual dose exposure, mGy | d_R_(y) | 1 | 3 | 6 | 10 | 8 | 6 | 4 | 2 |
| Annual dose < 5 mGy |  | 1 | 3 |  |  |  |  | 4 | 2 |
| Annual dose ≥ 5 mGy |  |  |  | 6 | 10 | 8 | 6 |  |  |
| Cumulative dose, mGy | d(y) | 1 | 4 | 10 | 20 | 28 | 34 | 38 | 40 |
| Cumulative dose received at annual dose rate <5mGy/year | d_L_(y) | 1 | 4 | 4 | 4 | 4 | 4 | 8 | 10 |
| Cumulative dose received at annual dose rate ≥5mGy/year | d_H_(y) | 0 | 0 | 6 | 16 | 24 | 30 | 30 | 30 |

1. Furuta H, Kudo S, Ishida J, Yoshimoto K, Kasagi F. Dose-rate effects on cancer mortality risk estimates for Japanese nuclear workers. In: Proceedings of the 2nd European Radiological Protection Research Week, Paris. 2017. Available at: http://www.rea.or.jp/ire/pdf/ 20171010Furuta.pdf. Accessed 23 August 2025

Table S3. Distribution of Mayak workers by accumulated gamma and neutron doses from external exposure and by cumulative alpha-particle doses from internal exposure

| Dose | Mayak Worker Cohort | Resident Subcohort (RSC) |
| --- | --- | --- |
| *Cumulative liver-absorbed gamma-ray dose (Gy)* | | |
| 0.00–0.10 | 8,673 (38.7%) | 5,078 (38.7%) |
| 0.10–0.20 | 3,327 (14.8%) | 2,107 (16%) |
| 0.20–0.50 | 4,382 (19.6%) | 2,543 (19.3%) |
| 0.50–0.75 | 1,669 (7.5%) | 947 (7.2%) |
| 0.75–1.00 | 1,129 (5%) | 651 (4.9%) |
| 1.00–1.50 | 1,520 (6.8%) | 865 (6.6%) |
| 1.50–2.00 | 866 (3.9%) | 499 (3.8%) |
| 2.00–3.00 | 641 (2.9%) | 393 (3%) |
| 3.00–4.00 | 130 (0.6%) | 55 (0.4%) |
| ≥4.00 | 40 (0.2%) | 17 (0.1%) |
| *Cumulative liver-absorbed neutron dose (Gy)* | | |
| Not measured | 18,294 (81.8%) | 10,573 (80.3%) |
| 0.00–0.0001 | 998 (4.5%) | 509 (3.8%) |
| 0.0001–0.001 | 2,025 (9%) | 1,261 (9.6%) |
| 0.001–0.0025 | 759 (3.4%) | 602 (4.6%) |
| 0.0025–0.005 | 174 (0.8%) | 125 (1%) |
| 0.005–0.01 | 74 (0.3%) | 50 (0.4%) |
| ≥0.01 | 53 (0.2%) | 36 (0.3%) |
| *Cumulative liver-absorbed alpha-particle dose from internal exposure (Gy)* | | |
| Not measured^a^ | 14,371 (64.2%) | 5,544 (42.1%) |
| 0–0.025 | 3,645 (16.3%) | 3,563 (27.1%) |
| 0.025–0.05 | 1,131 (5.1%) | 1,077 (8.2%) |
| 0.05–0.10 | 987 (4.4%) | 930 (7.1%) |
| 0.10–0.15 | 483 (2.2%) | 454 (3.5%) |
| 0.15–0.25 | 547 (2.4%) | 504 (3.7%) |
| 0.25–0.5 | 571 (2.6%) | 528 (4%) |
| 0.5–1.0 | 300 (1.3%) | 270 (2.1%) |
| ≥1.0 | 342 (1.5%) | 286 (2.2%) |
| *Notes:*  ^а^ percentage of the cohort (subcohort). | | |

Table S4. Excess relative risks of mortality from DCS in relation to the cumulative liver-absorbed gamma dose from external exposure, adjusted for different non-radiation factors and the liver-absorbed alpha-particle dose (sensitivity analysis: various lag periods, both sexes, entire cohort)

| Cut-off point  (Gy) | Model parameters | Lag periods | | | |
| --- | --- | --- | --- | --- | --- |
|  |  | 0 years | 5 years | 20 years | 30 years |
| Without a cut-off point | ERR/Gy | 0.03 (–0.02; 0.08) | 0.03 (–0.01; 0.09) | 0.04 (–0.01; 0.09) | 0.04 (–0.01; 0.10) |
| 0.005 | ERR_L_/Gy | **–5.39 (–6.59; –4.05)** | **–4.61 (–5.99; –3.09)** | **–3.41 (–5.57; –1.01)** | **–4.24 (–7.53; –0.57)** |
|  | ERR_H_/Gy | 0.02 (–0.02; 0.07) | 0.03 (–0.01; 0.08) | 0.03 (–0.01; 0.09) | 0.04 (–0.01; 0.10) |
|  | *p* value ^a^ | **< 0.001** | **< 0.001** | **0.006** | **0.024** |
| 0.010 | ERR_L_/Gy | **–2.52 (–3.11; –1.87)** | **–2.26 (–2.90; –1.56)** | **–1.99 (–2.85; –1.04)** | **–2.53 (–3.67; –1.27)** |
|  | ERR_H_/Gy | 0.03 (–0.01; 0.08) | 0.04 (–0.01; 0.09) | 0.04 (–0.01; 0.09) | 0.04 (–0.01; 0.10) |
|  | *p* value ^a^ | **< 0.001** | **< 0.001** | **< 0.001** | **< 0.001** |
| 0.015 | ERR_L_/Gy | **–1.74 (–2.16; –1.29)** | **–1.61 (–2.05; –1.13)** | **–1.43 (–1.99; –0.81)** | **–1.74 (–2.46; –0.94)** |
|  | ERR_H_/Gy | 0.04 (–0.00; 0.09) | 0.05 (–0.00; 0.10) | 0.04 (–0.00; 0.10) | 0.04 (–0.01; 0.10) |
|  | *p* value ^a^ | **< 0.001** | **< 0.001** | **< 0.001** | **< 0.001** |
| 0.020 | ERR_L_/Gy | **–1.29 (–1.61; –0.94)** | **–1.19 (–1.53; –0.82)** | **–1.06 (–1.48; –0.59)** | **–1.26 (–1.79; –0.68)** |
|  | ERR_H_/Gy | **0.05 (+0.00; 0.10)** | **0.05 (0.01; 0.10)** | **0.05 (+0.00; 0.10)** | **0.05 (+0.00; 0.11)** |
|  | *p* value ^a^ | **< 0.001** | **< 0.001** | **< 0.001** | **< 0.001** |
| 0.025 | ERR_L_/Gy | **–1.04 (–1.30; –0.75)** | **–0.96 (–1.23; –0.65)** | **–0.84 (–1.18; –0.47)** | **–1.07 (–1.48; –0.61)** |
|  | ERR_H_/Gy | **0.05 (0.01; 0.11)** | **0.06 (0.01; 0.11)** | **0.05 (+0.00; 0.11)** | **0.05 (+0.00; 0.11)** |
|  | *p* value ^a^ | **< 0.001** | **< 0.001** | **< 0.001** | **< 0.001** |
| 0.030 | ERR_L_/Gy | **–0.80 (–1.04; –0.55)** | **–0.73 (–0.98; –0.47)** | **–0.65 (–0.94; –0.33)** | **–0.83 (–1.18; –0.44)** |
|  | ERR_H_/Gy | **0.06 (0.01; 0.11)** | **0.06 (0.01; 0.11)** | **0.06 (0.01; 0.11)** | **0.06 (+0.00; 0.11)** |
|  | *p* value ^a^ | **< 0.001** | **< 0.001** | **< 0.001** | **< 0.001** |
| 0.035 | ERR_L_/Gy | **–0.63 (–0.83; –0.40)** | **–0.57 (–0.78; –0.33)** | **–0.50 (–0.76; –0.22)** | **–0.71 (–1.01; –0.38)** |
|  | ERR_H_/Gy | **0.06 (0.01; 0.11)** | **0.06 (0.01; 0.12)** | **0.06 (0.01; 0.11)** | **0.06 (+0.00; 0.12)** |
|  | *p* value ^a^ | **< 0.001** | **< 0.001** | **< 0.001** | **< 0.001** |
| 0.040 | ERR_L_/Gy | **–0.49 (–0.68; –0.29)** | **–0.44 (–0.63; –0.22)** | **–0.38 (–0.61; –0.13)** | **–0.55 (–0.82; –0.25)** |
|  | ERR_H_/Gy | **0.06 (0.01; 0.11)** | **0.06 (0.01; 0.12)** | **0.06 (0.01; 0.11)** | **0.06 (0.01; 0.12)** |
|  | *p* value ^a^ | **< 0.001** | **< 0.001** | **0.001** | **< 0.001** |
| 0.045 | ERR_L_/Gy | **–0.36 (–0.53; –0.17)** | **–0.31 (–0.49; –0.11)** | **–0.27 (–0.48; –0.04)** | **–0.42 (–0.67; –0.15)** |
|  | ERR_H_/Gy | **0.06 (0.01; 0.11)** | **0.06 (0.01; 0.12)** | **0.06 (+0.00; 0.11)** | **0.06 (+0.00; 0.12)** |
|  | *p* value ^a^ | **< 0.001** | **< 0.001** | **0.009** | **0.001** |
| 0.050 | ERR_L_/Gy | **–0.27 (–0.43; –0.08)** | **–0.23 (–0.40; –0.04)** | –0.17 (–0.37; 0.05) | **–0.33 (–0.56; –0.07)** |
|  | ERR_H_/Gy | **0.05 (+0.00; 0.11)** | **0.06 (+0.00; 0.11)** | **0.05 (+0.00; 0.11)** | **0.06 (+0.00; 0.12)** |
|  | *p* value ^a^ | **0.002** | **0.006** | **0.049** | **0.005** |
| *Notes:*  Values in bold are statistically significant estimates and differences;  The dataset was stratified by sex, attained age, calendar period, smoking status, alcohol consumption, and alpha-particle dose;  ERR/Gy denotes the excess relative risk per unit of radiation dose;  DCS denotes diseases of the circulatory system (ICD-9 codes: 390–459);  ^a^ Maximum likelihood test for comparison between models with and without a cut-off point. | | | | | |

Table S5. Excess relative risks of mortality from CeVD in relation to the cumulative liver-absorbed gamma dose from external exposure, adjusted for different non-radiation factors and liver-absorbed alpha-particle dose (sensitivity analysis: various lag periods, both sexes, entire cohort)

| Cut-off point (Gy) | Model parameters | Lag periods | | | |
| --- | --- | --- | --- | --- | --- |
|  |  | 0 years | 5 years | 20 years | 30 years |
| Without a cut-off point | ERR/Gy | 0.01 (–0.06; 0.11) | 0.03 (–0.05; 0.13) | 0.04 (–0.04; 0.15) | 0.06 (–0.03; 0.17) |
| 0.005 | ERR_L_/Gy | **–5.95 (n/a; –3.29)** | **–5.03 (–7.45; –2.09)** | –3.40 (–6.96; 0.89) | –4.75 (–9.74; 1.40) |
|  | ERR_H_/Gy | 0.02 (–0.06; 0.11) | 0.03 (–0.05; 0.12) | 0.04 (–0.04; 0.14) | 0.05 (–0.04; 0.16) |
|  | *p* value ^a^ | **< 0.001** | **0.002** | 0.108 | 0.117 |
| 0.010 | ERR_L_/Gy | **–2.40 (–3.45; –1.17)** | **–2.09 (–3.21; –0.78)** | –1.62 (–3.04; 0.07) | **–2.36 (–4.13; –0.23)** |
|  | ERR_H_/Gy | 0.03 (–0.05; 0.12) | 0.04 (–0.04; 0.14) | 0.05 (–0.03; 0.15) | 0.06 (–0.03; 0.17) |
|  | *p* value ^a^ | **< 0.001** | **0.003** | 0.053 | **0.028** |
| 0.015 | ERR_L_/Gy | **–1.71 (–2.44; –0.85)** | **–1.53 (–2.30; –0.63)** | **–1.25 (–2.18; –0.16)** | –1.34 (–2.53; 0.09) |
|  | ERR_H_/Gy | 0.03 (–0.04; 0.13) | 0.05 (–0.04; 0.14) | 0.06 (–0.03; 0.16) | 0.06 (–0.03; 0.17) |
|  | *p* value ^a^ | **< 0.001** | **0.001** | **0.022** | 0.054 |
| 0.020 | ERR_L_/Gy | **–1.30 (–1.86; –0.64)** | **–1.19 (–1.78; –0.49)** | **–0.91 (–1.62; –0.06)** | –0.88 (–1.77; 0.20) |
|  | ERR_H_/Gy | 0.04 (–0.04; 0.13) | 0.05 (–0.03; 0.15) | 0.06 (–0.03; 0.16) | 0.07 (–0.03; 0.18) |
|  | *p* value ^a^ | **< 0.001** | **0.001** | **0.029** | 0.084 |
| 0.025 | ERR_L_/Gy | **–1.10 (–1.55; –0.56)** | **–1.00 (–1.48; –0.44)** | **–0.87 (–1.42; –0.20)** | –0.81 (–1.50; 0.02) |
|  | ERR_H_/Gy | 0.05 (–0.03; 0.14) | 0.06 (–0.03; 0.15) | 0.06 (–0.02; 0.17) | 0.07 (–0.02; 0.18) |
|  | *p* value ^a^ | **< 0.001** | **< 0.001** | **0.008** | **0.04** |
| 0.030 | ERR_L_/Gy | **–0.88 (–1.27; –0.41)** | **–0.78 (–1.20; –0.28)** | **–0.69 (–1.17; –0.11)** | –0.63 (–1.23; 0.09) |
|  | ERR_H_/Gy | 0.05 (–0.03; 0.14) | 0.06 (–0.03; 0.16) | 0.07 (–0.02; 0.17) | 0.07 (–0.02; 0.18) |
|  | *p* value ^a^ | **< 0.001** | **0.002** | **0.014** | 0.056 |
| 0.035 | ERR_L_/Gy | **–0.77 (–1.11; –0.37)** | **–0.68 (–1.04; –0.25)** | **–0.63 (–1.04; –0.13)** | **–0.60 (–1.10; –0.00)** |
|  | ERR_H_/Gy | 0.05 (–0.03; 0.15) | 0.06 (–0.02; 0.16) | 0.07 (–0.02; 0.17) | 0.08 (–0.02; 0.19) |
|  | *p* value ^a^ | **< 0.001** | **0.002** | **0.009** | **0.03** |
| 0.040 | ERR_L_/Gy | **–0.62 (–0.93; –0.24)** | **–0.54 (–0.87; –0.13)** | **–0.50 (–0.87; –0.05)** | –0.46 (–0.91; 0.08) |
|  | ERR_H_/Gy | 0.05 (–0.03; 0.15) | 0.06 (–0.03; 0.16) | 0.07 (–0.02; 0.18) | 0.07 (–0.02; 0.19) |
|  | *p* value ^a^ | **0.002** | **0.007** | **0.019** | 0.056 |
| 0.045 | ERR_L_/Gy | **–0.47 (–0.76; –0.13)** | **–0.39 (–0.70; –0.02)** | –0.38 (–0.72; 0.03) | –0.39 (–0.79; 0.09) |
|  | ERR_H_/Gy | 0.05 (–0.04; 0.15) | 0.06 (–0.03; 0.16) | 0.07 (–0.02; 0.18) | 0.08 (–0.02; 0.19) |
|  | *p* value ^a^ | **0.007** | **0.025** | **0.04** | 0.063 |
| 0.050 | ERR_L_/Gy | **–0.35 (–0.62; –0.01)** | –0.27 (–0.56; 0.08) | –0.26 (–0.58; 0.14) | –0.30 (–0.66; 0.16) |
|  | ERR_H_/Gy | 0.04 (–0.04; 0.14) | 0.05 (–0.04; 0.15) | 0.06 (–0.03; 0.17) | 0.07 (–0.02; 0.19) |
|  | *p* value ^a^ | **0.031** | 0.084 | 0.114 | 0.109 |
| *Notes:*  Values in bold are statistically significant estimates and differences. The dataset was stratified by sex, attained age, calendar period, smoking status, alcohol consumption, and alpha-particle dose.  ERR/Gy denotes the excess relative risk per unit of radiation dose;  CeVD denotes cerebrovascular diseases (ICD-9 codes: 430–438);  n/a denotes the undefined boundary of the confidence interval;  ^a^ Maximum likelihood test for comparison between models with and without a cut-off point. | | | | | |

Table S6. Excess relative risks of mortality from IS in relation to the cumulative liver-absorbed gamma dose from external exposure, adjusted for different non-radiation factors and liver-absorbed alpha-particle dose (sensitivity analysis: various lag periods, both sexes, residents)

| Cut-off points (Gy) | Model parameters | Lag periods | | | |
| --- | --- | --- | --- | --- | --- |
|  |  | 0 years | 5 years | 20 years | 30 years |
| Without a cut-off point | ERR/Gy | 0.21 (–0.02; 0.54) | **0.33 (0.07; 0.71)** | 0.22 (–0.01; 0.57) | **0.27 (0.01; 0.64)** |
| 0.005 | ERR_L_/Gy | **–7.34 (n/a; –3.14)** | **–6.19 (na; –1.13)** | –5.53 (na; 1.92) | –9.10 (na; 1.98) |
|  | ERR_H_/Gy | 0.19 (–0.01; 0.48) | **0.31 (0.07; 0.65)** | 0.22 (–0.01; 0.54) | **0.26 (0.02; 0.60)** |
|  | *p* value ^a^ | **0.002** | **0.016** | 0.115 | 0.088 |
| 0.010 | ERR_L_/Gy | **–3.24 (–4.75; –1.12)** | **–2.96 (–4.62; –0.60)** | –2.80 (–5.03; 0.28) | –3.22 (na; 1.17) |
|  | ERR_H_/Gy | **0.24 (0.03; 0.54)** | **0.35 (0.11; 0.70)** | **0.24 (0.02; 0.57)** | **0.29 (0.04; 0.64)** |
|  | *p* value ^a^ | **0.003** | **0.009** | 0.053 | 0.106 |
| 0.015 | ERR_L_/Gy | **–2.40 (na; –0.88)** | **–2.30 (na; –0.65)** | –1.94 (–3.49; 0.13) | –2.04 (na; 0.83) |
|  | ERR_H_/Gy | **0.27 (0.05; 0.58)** | **0.38 (0.13; 0.74)** | **0.25 (0.02; 0.58)** | **0.29 (0.04; 0.65)** |
|  | *p* value ^a^ | **0.002** | **0.004** | **0.041** | 0.102 |
| 0.020 | ERR_L_/Gy | **–1.47 (–2.43; –0.17)** | **–1.45 (–2.47; –0.06)** | –1.08 (–2.33; 0.63) | –0.93 (–2.59; 1.44) |
|  | ERR_H_/Gy | **0.28 (0.05; 0.60)** | **0.39 (0.13; 0.76)** | **0.26 (0.02; 0.60)** | **0.29 (0.03; 0.65)** |
|  | *p* value ^a^ | **0.014** | **0.014** | 0.117 | 0.277 |
| 0.025 | ERR_L_/Gy | **–1.10 (–1.88; –0.01)** | –1.14 (–1.96; 0.02) | –0.85 (–1.85; 0.52) | –0.84 (–2.13; 0.98) |
|  | ERR_H_/Gy | **0.29 (0.05; 0.63)** | **0.41 (0.14; 0.79)** | **0.27 (0.03; 0.62)** | **0.30 (0.04; 0.67)** |
|  | *p* value ^a^ | **0.02** | **0.014** | 0.105 | 0.196 |
| 0.030 | ERR_L_/Gy | –0.76 (–1.43; 0.20) | –0.76 (–1.47; 0.26) | –0.53 (–1.37; 0.67) | –0.50 (–1.60; 1.08) |
|  | ERR_H_/Gy | **0.29 (0.05; 0.63)** | **0.41 (0.14; 0.80)** | **0.27 (0.02; 0.62)** | **0.30 (0.03; 0.67)** |
|  | *p* value ^a^ | **0.041** | **0.032** | 0.18 | 0.288 |
| 0.035 | ERR_L_/Gy | –0.73 (–1.29; 0.07) | –0.71 (–1.32; 0.16) | –0.56 (–1.25; 0.44) | –0.55 (–1.43; 0.72) |
|  | ERR_H_/Gy | **0.31 (0.06; 0.66)** | **0.44 (0.16; 0.84)** | **0.29 (0.03; 0.65)** | **0.32 (0.05; 0.69)** |
|  | *p* value ^a^ | **0.02** | **0.017** | 0.099 | 0.166 |
| 0.040 | ERR_L_/Gy | –0.63 (–1.15; 0.11) | –0.63 (–1.18; 0.15) | –0.48 (–1.11; 0.43) | –0.41 (–1.22; 0.75) |
|  | ERR_H_/Gy | **0.33 (0.07; 0.69)** | **0.46 (0.17; 0.87)** | **0.29 (0.04; 0.66)** | **0.32 (0.05; 0.70)** |
|  | *p* value ^a^ | **0.021** | **0.014** | 0.101 | 0.206 |
| 0.045 | ERR_L_/Gy | –0.43 (–0.91; 0.26) | –0.42 (–0.93; 0.32) | –0.27 (–0.86; 0.58) | –0.22 (–0.95; 0.83) |
|  | ERR_H_/Gy | **0.32 (0.06; 0.70)** | **0.47 (0.16; 0.89)** | **0.28 (0.02; 0.66)** | **0.32 (0.04; 0.70)** |
|  | *p* value ^a^ | **0.049** | **0.032** | 0.201 | 0.299 |
| 0.050 | ERR_L_/Gy | –0.32 (–0.79; 0.34) | –0.31 (–0.80; 0.39) | –0.17 (–0.73; 0.64) | –0.10 (–0.78; 0.89) |
|  | ERR_H_/Gy | **0.32 (0.05; 0.70)** | **0.47 (0.16; 0.90)** | **0.28 (0.02; 0.66)** | **0.31 (0.03; 0.71)** |
|  | *p* value ^a^ | 0.076 | **0.046** | 0.269 | 0.391 |
| *Notes:*  Values in bold are statistically significant estimates and differences. The dataset was stratified by sex, attained age, calendar period, smoking status, alcohol consumption, and alpha-particle dose.  ERR/Gy denotes the excess relative risk per unit of radiation dose;  IS denotes ischemic stroke (ICD-9 codes: 434);  n/a denotes the undefined boundary of the confidence interval;  ^a^ Maximum likelihood test for comparison between models with and without a cut-off point. | | | | | |

Table S7. Excess relative risks of mortality from DCS in relation to the cumulative 10-year lagged liver-absorbed gamma dose from external exposure (sensitivity analysis: various adjustments for alpha-particle and neutron doses, period and age at hire; both sexes, entire cohort)

| Cut-off point (Gy) | Model parameters | Sensitivity analysis type | | | |
| --- | --- | --- | --- | --- | --- |
|  |  | Removing the adjustment for alpha dose | Association with the weighted sum of gamma and neutron doses (Sv) ^b^ | Adjusting for the period of hire | Adjusting for age at hire |
| Without the cut-off point | ERR/Gy | 0.02 (–0.02; 0.07) | 0.03 (–0.02; 0.08) | **0.06 (+0.00; 0.12)** | **0.08 (0.03; 0.15)** |
| 0.005 | ERR_L_/Gy | **–4.53**  **(–5.95; –2.96)** | **–4.14**  **(–5.70; –2.41)** | **–4.39**  **(–5.96; –2.63)** | **–4.56**  **(–6.18; –2.76)** |
|  | ERR_H_/Gy | 0.02 (–0.02; 0.06) | 0.03 (–0.02; 0.08) | **0.05 (+0.00; 0.11)** | **0.08 (0.02; 0.14)** |
|  | *p* value ^c^ | **< 0.001** | **< 0.001** | **< 0.001** | **< 0.001** |
| 0.010 | ERR_L_/Gy | **–2.12**  **(–2.78; –1.40)** | **–2.11**  **(–2.80; –1.36)** | **–2.28**  **(–2.97; –1.51)** | **–2.31**  **(–3.03; –1.52)** |
|  | ERR_H_/Gy | 0.03 (–0.01; 0.07) | 0.04 (–0.01; 0.09) | **0.06 (0.01; 0.12)** | **0.09 (0.03; 0.15)** |
|  | *p* value ^c^ | **< 0.001** | **< 0.001** | **< 0.001** | **< 0.001** |
| 0.015 | ERR_L_/Gy | **–1.43**  **(–1.89; –0.94)** | **–1.54**  **(–2.00; –1.03)** | **–1.62**  **(–2.09; –1.10)** | **–1.69**  **(–2.17; –1.16)** |
|  | ERR_H_/Gy | 0.03 (–0.01; 0.07) | 0.04 (–0.01; 0.09) | **0.07 (0.01; 0.12)** | **0.09 (0.04; 0.15)** |
|  | *p* value ^c^ | **< 0.001** | **< 0.001** | **< 0.001** | **< 0.001** |
| 0.020 | ERR_L_/Gy | **–0.99**  **(–1.34; –0.60)** | **–1.12**  **(–1.48; –0.74)** | **–1.23**  **(–1.59; –0.83)** | **–1.30**  **(–1.67; –0.89)** |
|  | ERR_H_/Gy | 0.03 (–0.01; 0.08) | 0.05 (–0.00; 0.10) | **0.07 (0.02; 0.13)** | **0.10 (0.04; 0.16)** |
|  | *p* value ^c^ | **< 0.001** | **< 0.001** | **< 0.001** | **< 0.001** |
| 0.025 | ERR_L_/Gy | **–0.72**  **(–1.02; –0.40)** | **–0.90**  **(–1.19; –0.58)** | **–0.98**  **(–1.28; –0.66)** | **–1.04**  **(–1.34; –0.70)** |
|  | ERR_H_/Gy | 0.03 (–0.01; 0.08) | **0.05 (+0.00; 0.10)** | **0.08 (0.02; 0.14)** | **0.11 (0.05; 0.17)** |
|  | *p* value ^c^ | **< 0.001** | **< 0.001** | **< 0.001** | **< 0.001** |
| 0.030 | ERR_L_/Gy | **–0.52**  **(–0.77; –0.24)** | **–0.72**  **(–0.97; –0.44)** | **–0.77**  **(–1.03; –0.49)** | **–0.83**  **(–1.10; –0.54)** |
|  | ERR_H_/Gy | 0.04 (–0.01; 0.08) | **0.05 (0.01; 0.11)** | **0.08 (0.03; 0.14)** | **0.11 (0.05; 0.18)** |
|  | *p* value ^c^ | **< 0.001** | **< 0.001** | **< 0.001** | **< 0.001** |
| 0.035 | ERR_L_/Gy | **–0.36**  **(–0.59; –0.12)** | **–0.55**  **(–0.77; –0.30)** | **–0.59**  **(–0.82; –0.33)** | **–0.65**  **(–0.88; –0.39)** |
|  | ERR_H_/Gy | 0.04 (–0.01; 0.08) | **0.06 (0.01; 0.11)** | **0.08 (0.03; 0.14)** | **0.12 (0.06; 0.18)** |
|  | *p* value ^c^ | **0.003** | **< 0.001** | **< 0.001** | **< 0.001** |
| 0.040 | ERR_L_/Gy | **–0.25**  **(–0.46; –0.03)** | **–0.42**  **(–0.62; –0.20)** | **–0.46**  **(–0.67; –0.23)** | **–0.52**  **(–0.73; –0.28)** |
|  | ERR_H_/Gy | 0.03 (–0.01; 0.08) | **0.06 (0.01; 0.11)** | **0.08 (0.03; 0.15)** | **0.12 (0.06; 0.19)** |
|  | *p* value ^c^ | **0.014** | **< 0.001** | **< 0.001** | **< 0.001** |
| 0.045 | ERR_L_/Gy | –0.16  (–0.35; 0.05) | **–0.30**  **(–0.48; –0.09)** | **–0.33**  **(–0.52; –0.11)** | **–0.38**  **(–0.58; –0.17)** |
|  | ERR_H_/Gy | 0.03 (–0.01; 0.08) | **0.05 (+0.00; 0.11)** | **0.08 (0.03; 0.15)** | **0.12 (0.06; 0.19)** |
|  | *p* value ^c^ | 0.073 | **0.002** | **< 0.001** | **< 0.001** |
| 0.050 | ERR_L_/Gy | –0.10  (–0.27; 0.10) | **–0.21**  **(–0.39; –0.01)** | **–0.23**  **(–0.42; –0.03)** | **–0.28**  **(–0.47; –0.07)** |
|  | ERR_H_/Gy | 0.03 (–0.01; 0.08) | **0.05 (+0.00; 0.11)** | **0.08 (0.02; 0.14)** | **0.11 (0.05; 0.18)** |
|  | *p* value ^c^ | 0.202 | **0.014** | **0.005** | **< 0.001** |
| *Notes:*  Values in bold are statistically significant estimates and differences;  The dataset was stratified by sex, attained age, calendar period, smoking status, alcohol consumption, and alpha-particle dose;  ERR/Gy denotes the excess relative risk per unit of radiation dose;  DCS denotes diseases of the circulatory system (ICD-9 codes: 390–459);  ^a^ Workers without bioassay measurements of alpha activity were divided into two groups: reactor personnel and unmonitored workers.  ^b^ The dataset included all workers.  ^c^ Maximum likelihood test for comparison between models with and without a cut-off point. | | | | | |

Table S8. Excess relative risks of mortality from CeVD in relation to the cumulative 10-year lagged liver-absorbed gamma dose from external exposure (sensitivity analysis: various adjustments for alpha-particle and neutron doses, period and age at hire; both sexes, entire cohort)

| Cut-off point (Gy) | Model parameters | Type of the sensitivity analysis | | | |
| --- | --- | --- | --- | --- | --- |
|  |  | Removing the alpha dose adjustment | Association with the weighted sum of gamma and neutron doses (Sv) ^b^ | Adjusting for the period of hire | Adjusting for age at hire |
| Without a cut-off point | ERR/Gy | +0.00 (–0.06; 0.09) | 0.03 (–0.05; 0.13) | 0.09 (–0.01; 0.21) | **0.11 (0.01; 0.24)** |
| 0.005 | ERR_L_/Gy | **–4.81**  **(–7.24; –1.89)** | **–4.47**  **(–7.17; –1.20)** | **–4.83**  **(–7.54; –1.53)** | **–4.99**  **(–7.76; –1.54)** |
|  | ERR_H_/Gy | 0.01 (–0.06; 0.09) | 0.03 (–0.05; 0.13) | 0.09 (–0.01; 0.21) | **0.11 (0.01; 0.24)** |
|  | *p* value ^c^ | **0.002** | **0.009** | **0.005** | **0.006** |
| 0.010 | ERR_L_/Gy | **–1.86**  **(–2.99; –0.55)** | **–2.02**  **(–3.17; –0.65)** | **–2.16**  **(–3.35; –0.75)** | **–2.11**  **(–3.35; –0.63)** |
|  | ERR_H_/Gy | 0.01 (–0.05; 0.09) | 0.04 (–0.04; 0.14) | **0.10 (+0.00; 0.22)** | **0.12 (0.02; 0.25)** |
|  | *p* value ^c^ | **0.007** | **0.005** | **0.003** | **0.005** |
| 0.015 | ERR_L_/Gy | **–1.29**  **(–2.07; –0.37)** | **–1.57**  **(–2.34; –0.65)** | **–1.59**  **(–2.41; –0.62)** | **–1.60**  **(–2.46; –0.58)** |
|  | ERR_H_/Gy | 0.02 (–0.05; 0.10) | 0.05 (–0.03; 0.15) | **0.10 (0.01; 0.22)** | **0.13 (0.02; 0.25)** |
|  | *p* value ^c^ | **0.007** | **0.001** | **0.001** | **0.002** |
| 0.020 | ERR_L_/Gy | **–0.84**  **(–1.47; –0.10)** | **–1.14**  **(–1.74; –0.42)** | **–1.16**  **(–1.81; –0.39)** | **–1.17**  **(–1.85; –0.36)** |
|  | ERR_H_/Gy | 0.02 (–0.05; 0.10) | 0.05 (–0.03; 0.15) | **0.11 (0.01; 0.22)** | **0.13 (0.03; 0.26)** |
|  | *p* value ^c^ | **0.026** | **0.002** | **0.003** | **0.003** |
| 0.025 | ERR_L_/Gy | **–0.66**  **(–1.17; –0.06)** | **–1.01**  **(–1.50; –0.43)** | **–0.96**  **(–1.49; –0.33)** | **–0.96**  **(–1.51; –0.29)** |
|  | ERR_H_/Gy | 0.02 (–0.05; 0.10) | 0.06 (–0.03; 0.16) | **0.11 (0.01; 0.23)** | **0.14 (0.03; 0.27)** |
|  | *p* value ^c^ | **0.031** | **< 0.001** | **0.002** | **0.003** |
| 0.030 | ERR_L_/Gy | –0.46  (–0.91; 0.07) | **–0.82**  **(–1.24; –0.31)** | **–0.76**  **(–1.22; –0.20)** | **–0.75**  **(–1.23; –0.16)** |
|  | ERR_H_/Gy | 0.02 (–0.05; 0.10) | 0.06 (–0.02; 0.16) | **0.11 (0.01; 0.24)** | **0.14 (0.03; 0.27)** |
|  | *p* value ^c^ | 0.081 | **0.002** | **0.004** | **0.005** |
| 0.035 | ERR_L_/Gy | –0.41  (–0.80; 0.05) | **–0.73**  **(–1.10; –0.28)** | **–0.65**  **(–1.05; –0.17)** | **–0.64**  **(–1.06; –0.12)** |
|  | ERR_H_/Gy | 0.02 (–0.05; 0.10) | 0.07 (–0.02; 0.17) | **0.12 (0.02; 0.24)** | **0.14 (0.04; 0.27)** |
|  | *p* value ^c^ | 0.073 | **0.001** | **0.004** | **0.006** |
| 0.040 | ERR_L_/Gy | –0.27  (–0.63; 0.15) | **–0.57**  **(–0.90; –0.16)** | **–0.50**  **(–0.87; –0.05)** | –0.48  (–0.87; +0.00) |
|  | ERR_H_/Gy | 0.02 (–0.06; 0.10) | 0.07 (–0.02; 0.17) | **0.12 (0.01; 0.24)** | **0.14 (0.03; 0.28)** |
|  | *p* value ^c^ | 0.175 | **0.004** | **0.011** | **0.016** |
| 0.045 | ERR_L_/Gy | –0.18  (–0.50; 0.21) | **–0.39**  **(–0.70; –0.01)** | –0.37  (–0.70; 0.05) | –0.33  (–0.69; 0.12) |
|  | ERR_H_/Gy | 0.01 (–0.06; 0.10) | 0.06 (–0.03; 0.17) | **0.11 (0.01; 0.24)** | **0.14 (0.03; 0.27)** |
|  | *p* value ^c^ | 0.32 | **0.028** | **0.03** | **0.046** |
| 0.050 | ERR_L_/Gy | –0.09  (–0.40; 0.28) | –0.29  (–0.59; 0.07) | –0.22  (–0.55; 0.18) | –0.19  (–0.53; 0.24) |
|  | ERR_H_/Gy | 0.01 (–0.06; 0.09) | 0.06 (–0.03; 0.16) | **0.11 (+0.00; 0.23)** | **0.13 (0.02; 0.27)** |
|  | *p* value ^c^ | > 0.50 | 0.07 | 0.115 | 0.144 |
| *Notes:*  Values in bold are statistically significant estimates and differences;  ERR/Gy denotes the excess relative risk per unit of radiation dose;  CeVD denotes cerebrovascular diseases (ICD-9 codes: 430–438);  ^a^ Workers without bioassay measurements of alpha activity were divided into two groups: reactor personnel and unmonitored workers.  ^b^ The dataset considered all workers.  ^c^ Maximum likelihood test for comparison between models with and without a cut-off point. | | | | | |

Table S9. Excess relative risks of mortality from IS in relation to the cumulative 10-year lagged liver-absorbed gamma dose from external exposure (sensitivity analysis: various adjustments for alpha-particle and neutron doses, period and age at hire; both sexes, residents)

| Cut-off point (Gy) | Model parameters | Type of the sensitivity analysis | | | |
| --- | --- | --- | --- | --- | --- |
|  |  | Removing the alpha dose adjustment | Association with the weighted sum of gamma and neutron doses (Sv) ^b^ | Adjusting for the period of hire | Adjusting for age at hire |
| Without a cut-off point | ERR/Gy | 0.10 (–0.06; 0.33) | 0.22 (–0.01; 0.56) | **0.39 (0.08; 0.89)** | **0.42 (0.10; 0.93)** |
| 0.005 | ERR_L_/Gy | **–6.24**  **(n/a; –1.59)** | –5.37  (na; 0.44) | –5.09  (–9.16; 1.09) | –5.38  (na; 0.86) |
|  | ERR_H_/Gy | 0.10 (–0.05; 0.30) | **0.22 (+0.00; 0.53)** | **0.38 (0.08; 0.85)** | **0.39 (0.09; 0.86)** |
|  | *p* value ^c^ | **0.011** | 0.057 | 0.076 | 0.064 |
| 0.010 | ERR_L_/Gy | **–2.49**  **(–4.21; –0.15)** | –2.51  (–4.33; 0.03) | –2.42  (–4.40; 0.41) | –2.32  (–4.37; 0.64) |
|  | ERR_H_/Gy | 0.12 (–0.04; 0.33) | **0.24 (0.02; 0.56)** | **0.41 (0.11; 0.88)** | **0.42 (0.12; 0.90)** |
|  | *p* value ^c^ | **0.032** | **0.036** | **0.049** | 0.064 |
| 0.015 | ERR_L_/Gy | –1.55  (–2.88; 0.20) | **–2.09**  **(–3.40; –0.34)** | –1.93  (–3.38; 0.07) | **–2.09**  **(–3.52; –0.09)** |
|  | ERR_H_/Gy | 0.13 (–0.04; 0.34) | **0.27 (0.04; 0.59)** | **0.43 (0.13; 0.91)** | **0.44 (0.14; 0.92)** |
|  | *p* value ^c^ | 0.061 | **0.012** | **0.024** | **0.017** |
| 0.020 | ERR_L_/Gy | –0.47  (–1.66; 1.12) | –1.18  (–2.27; 0.29) | –1.01  (–2.24; 0.72) | –1.21  (–2.39; 0.49) |
|  | ERR_H_/Gy | 0.11 (–0.05; 0.34) | **0.27 (0.04; 0.60)** | **0.44 (0.12; 0.93)** | **0.45 (0.14; 0.94)** |
|  | *p* value ^c^ | 0.434 | 0.056 | 0.093 | 0.053 |
| 0.025 | ERR_L_/Gy | –0.09  (–1.12; 1.29) | –0.85  (–1.75; 0.40) | –0.68  (–1.70; 0.79) | –0.83  (–1.82; 0.63) |
|  | ERR_H_/Gy | 0.11 (–0.06; 0.34) | **0.28 (0.04; 0.62)** | **0.45 (0.12; 0.95)** | **0.47 (0.14; 0.97)** |
|  | *p* value ^c^ | > 0.50 | 0.078 | 0.122 | 0.075 |
| 0.030 | ERR_L_/Gy | 0.17  (–0.74; 1.40) | –0.59  (–1.35; 0.50) | –0.36  (–1.25; 0.94) | –0.47  (–1.33; 0.83) |
|  | ERR_H_/Gy | 0.10 (–0.07; 0.33) | **0.29 (0.04; 0.63)** | **0.45 (0.12; 0.96)** | **0.47 (0.14; 0.99)** |
|  | *p* value ^c^ | > 0.50 | 0.113 | 0.196 | 0.135 |
| 0.035 | ERR_L_/Gy | 0.11  (–0.66; 1.17) | –0.56  (–1.21; 0.35) | –0.44  (–1.18; 0.65) | –0.52  (–1.23; 0.56) |
|  | ERR_H_/Gy | 0.10 (–0.07; 0.34) | **0.30 (0.05; 0.66)** | **0.48 (0.14; 1.00)** | **0.50 (0.16; 1.03)** |
|  | *p* value ^c^ | > 0.50 | 0.071 | 0.094 | 0.062 |
| 0.040 | ERR_L_/Gy | 0.17  (–0.54; 1.14) | –0.50  (–1.08; 0.31) | –0.40  (–1.06; 0.59) | –0.47  (–1.12; 0.50) |
|  | ERR_H_/Gy | 0.10 (–0.08; 0.33) | **0.32 (0.06; 0.68)** | **0.49 (0.15; 1.03)** | **0.52 (0.17; 1.06)** |
|  | *p* value ^c^ | > 0.50 | 0.061 | 0.081 | **0.05** |
| 0.045 | ERR_L_/Gy | 0.27  (–0.38; 1.16) | –0.27  (–0.81; 0.51) | –0.19  (–0.82; 0.74) | –0.27  (–0.88; 0.66) |
|  | ERR_H_/Gy | 0.09 (–0.09; 0.32) | **0.30 (0.04; 0.67)** | **0.49 (0.14; 1.04)** | **0.52 (0.17; 1.07)** |
|  | *p* value ^c^ | > 0.50 | 0.158 | 0.147 | 0.092 |
| 0.050 | ERR_L_/Gy | 0.33  (–0.28; 1.18) | –0.19  (–0.71; 0.54) | –0.08  (–0.68; 0.83) | –0.16  (–0.74; 0.73) |
|  | ERR_H_/Gy | 0.08 (–0.09; 0.32) | **0.30 (0.04; 0.68)** | **0.48 (0.13; 1.04)** | **0.52 (0.16; 1.08)** |
|  | *p* value ^c^ | 0.486 | 0.192 | 0.21 | 0.126 |
| *Notes:*  Values in bold are statistically significant estimates and differences;  ERR/Gy denotes the excess relative risk per unit of radiation dose;  IS denotes ischemic stroke (ICD-9 code 434);  n/a denotes the undefined boundary of the confidence interval;  ^a^ Workers without bioassay measurements of alpha activity were divided into two groups: reactor personnel and unmonitored workers.  ^b^ The dataset considered all workers.  ^c^ Maximum likelihood test for comparison between models with and without a cut-off point. | | | | | |
